# Brain Neuroplasticity Leveraging Virtual Reality and Brain–Computer Interface Technologies

**DOI:** 10.3390/s24175725

**Published:** 2024-09-03

**Authors:** Athanasios Drigas, Angeliki Sideraki

**Affiliations:** 1 Net Media Lab & Mind & Brain R&D, Institute of Informatics & Telecommunications, National Centre of Scientific Research ‘Demokritos’, 15341 Athens, Greece; 2 Department of Secondary Education, Kapodistrian University of Athens, 15772 Athens, Greece; angeside@ppp.uoa.gr

**Keywords:** neuroplasticity, virtual reality, brain, brain–computer interface—BCI

## Abstract

This study explores neuroplasticity through the use of virtual reality (VR) and brain–computer interfaces (BCIs). Neuroplasticity is the brain’s ability to reorganize itself by forming new neural connections in response to learning, experience, and injury. VR offers a controlled environment to manipulate sensory inputs, while BCIs facilitate real-time monitoring and modulation of neural activity. By combining VR and BCI, researchers can stimulate specific brain regions, trigger neurochemical changes, and influence cognitive functions such as memory, perception, and motor skills. Key findings indicate that VR and BCI interventions are promising for rehabilitation therapies, treatment of phobias and anxiety disorders, and cognitive enhancement. Personalized VR experiences, adapted based on BCI feedback, enhance the efficacy of these interventions. This study underscores the potential for integrating VR and BCI technologies to understand and harness neuroplasticity for cognitive and therapeutic applications. The researchers utilized the PRISMA (Preferred Reporting Items for Systematic Reviews and Meta-Analyses) method to conduct a comprehensive and systematic review of the existing literature on neuroplasticity, VR, and BCI. This involved identifying relevant studies through database searches, screening for eligibility, and assessing the quality of the included studies. Data extraction focused on the effects of VR and BCI on neuroplasticity and cognitive functions. The PRISMA method ensured a rigorous and transparent approach to synthesizing evidence, allowing the researchers to draw robust conclusions about the potential of VR and BCI technologies in promoting neuroplasticity and cognitive enhancement.

## 1. Introduction

The human brain is a remarkably adaptable organ, capable of undergoing structural and functional changes in response to environmental stimuli and experiential learning [1]. The phenomenon, known as neuroplasticity, forms the foundation of our ability to acquire new skills, form memories, and adapt to changing circumstances throughout life. Neuroplasticity encompasses a broad spectrum of mechanisms, including synaptic plasticity, dendritic remodeling, and changes in neural connectivity, all of which contribute to the brain’s dynamic capacity for reorganization [2]. In recent years, advances in technology have provided unprecedented opportunities to explore and manipulate neuroplastic processes. One such technology that has garnered significant attention is virtual reality (VR). VR immerses users in computer-generated environments that simulate real-world experiences, engaging multiple sensory modalities and providing a rich and interactive sensory experience [3]. By manipulating the sensory inputs received by the brain, VR has the potential to induce neural changes and facilitate adaptive responses within the central nervous system. The use of VR as a tool for studying neuroplasticity offers several advantages over traditional experimental approaches. Unlike conventional laboratory tasks, VR environments can be tailored to provide highly controlled and customizable stimuli, allowing researchers to systematically manipulate sensory inputs and cognitive demands [4]. Moreover, VR enables researchers to create immersive experiences that closely mimic real-world scenarios, enhancing ecological validity and promoting more naturalistic patterns of brain activity.

Virtual reality (VR) technology has made significant strides in recent years, marking substantial advancements and widespread applications across various fields.

Social VR platforms have seen significant growth, enabling real-time interactions in virtual environments. These platforms facilitate activities such as virtual meetups, concerts, and collaborative workspaces, enhancing social connections and creating immersive shared experiences. This trend is making VR more inclusive and community-focused [5].

In healthcare, VR is revolutionizing patient care and medical training. VR-based therapies have proven effective for treating mental health conditions such as anxiety, Post-Traumatic Stress Disorder (PTSD0, and phobias through immersive exposure therapy. Additionally, VR is utilized for physical rehabilitation, allowing patients to engage in interactive exercises with real-time feedback, which significantly aids in recovery processes. Studies show that VR helps stroke patients practice and relearn daily activities, providing a new dimension to rehabilitation [6]. Moreover, institutions like Johnson & Johnson are using VR to train surgeons, improving surgical skills and patient outcomes through realistic simulations [7].

In the business sector, VR is enhancing training programs, facilitating remote collaboration, and improving design and prototyping processes. Companies are leveraging VR for staff training and customer service, showcasing its versatility beyond gaming and entertainment. The integration of artificial intelligence (AI) into VR creates personalized and adaptive virtual environments, making interactions more intuitive and efficient [8].

Moreover, VR is transforming education by creating immersive learning experiences that cater to diverse learning styles. Virtual field trips, interactive simulations, and augmented reality (AR) integrations make complex concepts more accessible and engaging. This democratization of education enables students worldwide to benefit from high-quality, immersive learning environments [9]. Moreover, the development of the Metaverse is set to redefine VR by creating interconnected virtual worlds where users can engage in a wide range of activities. This digital universe aims to provide a continuous, immersive experience that integrates work, learning, and play, representing a significant shift in how digital content is consumed and interacted [10].

Also, the convergence of VR and AR technologies is giving rise to mixed-reality experiences that blend digital content with the real world. This fusion enhances various applications, from retail and advertising to navigation and remote assistance, offering a seamless interaction between users and their environments [11].

At the same time, brain–computer interface (BCI) is a system that enables direct communication between the brain and external devices, allowing for real-time monitoring and modulation of neural activity. By leveraging BCIs, researchers and clinicians can gain unprecedented insights into brain function and dynamically influence neural processes. This capability is particularly significant for neuroplasticity, as it allows for precise, individualized interventions that can promote beneficial neural changes [12].

One of the key areas where BCIs have shown promise is in the rehabilitation of motor functions following neurological injuries such as stroke [13]. Stroke often results in significant motor deficits due to damage to specific brain regions. Traditional rehabilitation techniques rely on repetitive physical exercises to stimulate neural plasticity and recovery [14]. However, these methods can be enhanced by integrating BCI technology. For instance, BCIs can be used to detect motor intention in stroke patients and translate these intentions into movements of a robotic limb or virtual avatar, providing immediate feedback and reinforcing the neural pathways involved in motor control [15]. This closed-loop system can accelerate motor recovery by continuously adapting the rehabilitation process to the patient’s neural responses [16].

BCIs also hold potential in the treatment of cognitive and emotional disorders. For example, in the context of anxiety and phobias, BCIs can be used to monitor neural markers of anxiety and modulate virtual reality (VR) environments in real time to help patients confront and manage their fears in a controlled setting [17]. By providing tailored exposures and adjusting difficulty levels based on real-time neural feedback, BCIs can enhance the efficacy of exposure therapy and promote long-lasting neural adaptations that reduce anxiety symptoms [18].

In addition to rehabilitation and therapy, BCIs offer promising applications for cognitive enhancement in healthy individuals. By facilitating neurofeedback training, where individuals learn to regulate their own brain activity, BCIs can improve cognitive functions such as attention, memory, and executive function. Studies have shown that neurofeedback can induce neuroplastic changes in the brain, leading to improved cognitive performance [19]. For instance, training individuals to increase the amplitude of certain brain wave frequencies can enhance attention and working memory, with corresponding changes observed in neural connectivity patterns [20].

The integration of BCIs with VR technology further amplifies their potential. VR provides immersive environments that can be precisely controlled and manipulated to engage various cognitive and motor processes. When combined with BCI, VR can create interactive scenarios that adapt in real time to the user’s brain activity, optimizing the engagement and effectiveness of the intervention. This approach has been explored in several studies, demonstrating enhanced motor learning and cognitive training outcomes compared to traditional methods [21].

Despite promising advances, the implementation of BCIs in clinical and everyday settings faces several challenges. Ensuring the accuracy and reliability of BCIs, managing the complexity of neural signals, and addressing ethical considerations related to privacy and autonomy are critical areas that require ongoing research and development. Additionally, the cost and accessibility of BCI technology need to be addressed to make these interventions widely available [22].

Moreover, BCIs represent a transformative tool for harnessing neuroplasticity in various applications, from rehabilitation and therapy to cognitive enhancement and research. By enabling precise and adaptive modulation of brain activity, BCIs can facilitate targeted interventions that promote beneficial neural changes. As research and technology continue to advance, the integration of BCIs with other emerging technologies like VR holds the promise of unlocking new dimensions of brain health and resilience. The ongoing exploration of the BCI’s potential will undoubtedly lead to innovative strategies for enhancing neuroplasticity and improving the quality of life for individuals across a wide spectrum of needs [23].

A comprehensive literature search was conducted using the Preferred Reporting Items for Systematic Reviews and Meta-Analyses (PRISMA) guidelines to identify relevant studies examining the intersection of neuroplasticity, VR, BCIs, and cognitive enhancement. The search was performed across multiple electronic databases, including PubMed, Web of Science, Scopus, and PsycINFO. The search terms combined keywords and MeSH terms related to neuroplasticity, virtual reality, brain–computer interfaces, cognitive enhancement, rehabilitation, and specific cognitive functions (e.g., memory, attention, executive function). The search strategy included terms such as “neuroplasticity”, “virtual reality”, “VR”, “brain omputer interface”, “BCI”, “cognitive enhancement”, “rehabilitation”, “memory”, “attention”, and “executive function”.

In the following sections, we will review the existing literature on neuroplasticity and VR, describe the methodology employed in our study, present our findings, and discuss their implications for our understanding of brain plasticity and the potential applications of VR technology in neuroscience and clinical practice.

This study is critically important for science due to its comprehensive exploration of the intersections between neuroplasticity, virtual reality (VR), brain–computer interfaces (BCIs), and cognitive enhancement. Here are several key reasons why this study contributes significantly to scientific knowledge and application:

Advancing Understanding of Neuroplasticity: By delving into how VR and BCIs can induce neural changes, this study enhances our understanding of neuroplasticity—the brain’s ability to reorganize and adapt in response to stimuli and experiences. This foundational knowledge is crucial for developing therapies that leverage the brain’s adaptive capabilities for rehabilitation and cognitive enhancement. Also, this study highlights VR’s transformative role in healthcare, from mental health treatments like exposure therapy for anxiety and PTSD to physical rehabilitation for stroke patients. VR’s immersive nature allows for interactive exercises and simulations that enhance patient engagement and improve outcomes, demonstrating its potential to revolutionize medical care.

In the education and business sectors, VR is shown to create immersive learning experiences and enhance training programs. This innovation democratizes education by making complex concepts more accessible and engaging, while in business, it facilitates remote collaboration and improves prototyping processes. This study underscores VR’s versatility beyond entertainment, fostering advancements in diverse fields.

By integrating BCIs with VR, this study explores personalized interventions that dynamically adapt to neural activity in real-time. This approach optimizes therapeutic effectiveness in rehabilitation and enhances cognitive training outcomes. It also addresses challenges such as accuracy, reliability, and ethical considerations, paving the way for the responsible deployment of these technologies in clinical and everyday settings.

This study’s adherence to PRISMA guidelines ensures methodological rigor in reviewing the existing literature across multiple databases. This systematic approach synthesizes current research on neuroplasticity, VR, BCIs, and cognitive enhancement, providing a comprehensive overview that informs future studies and applications. Ultimately, this study contributes to advancing brain health and resilience by exploring innovative strategies that harness neuroplasticity. By enabling precise modulation of brain activity through VR and BCIs, this study opens avenues for targeted interventions that improve quality of life across various needs—from rehabilitation to cognitive enhancement.

## 2. Materials and Methods

To ensure a thorough and transparent methodology in our systematic review and meta-analysis on the use of virtual reality (VR) and brain–computer interfaces (BCIs) in harnessing neuroplasticity for cognitive enhancement and rehabilitation, we adhered to the Preferred Reporting Items for Systematic Reviews and Meta-Analyses (PRISMA) guidelines. This approach provided a rigorous framework for the systematic identification, selection, and critical evaluation of relevant research studies, thereby enhancing the reproducibility, reliability, and credibility of our findings.

### 2.1. Search Strategy and Information Sources

A comprehensive and systematic search was conducted across several major electronic databases, including PubMed, PsycINFO, Web of Science, and IEEE Xplore, to identify relevant studies published up to June 2024. The search strategy involved the use of a detailed set of keywords and Medical Subject Headings (MeSH) terms that were designed to capture the intersection of neuroplasticity, VR, BCIs, cognitive enhancement, and rehabilitation. The specific search terms employed were the following:

“neuroplasticity” AND “virtual reality”
“neuroplasticity” AND “brain computer interface”
“VR” AND “cognitive rehabilitation”
“BCI” AND “motor recovery”
“virtual reality” AND “neurofeedback”
“brain computer interface” AND “memory enhancement”

Boolean operators were utilized to combine these terms effectively, enhancing the precision and breadth of the search. Additionally, database-specific filters were applied to limit the results to peer-reviewed journal articles and conference proceedings published in English, ensuring a high standard of scientific quality and relevance.

### 2.2. Inclusion and Exclusion Criteria

To establish a robust and comprehensive overview of the research landscape, we defined the specific inclusion and exclusion criteria:

### 2.3. Inclusion Criteria

Studies involving human participants to ensure direct applicability to human neuroplasticity and cognitive functions.

Research employing VR or BCI interventions specifically aimed at modulating neuroplasticity, including studies that used VR for immersive simulations or BCIs for direct brain–computer communication.

Articles that investigated cognitive enhancement or rehabilitation outcomes, such as improvements in memory, executive functions, or motor skills.

Publications appearing in peer-reviewed journals or presented at reputable academic conferences, ensuring that the studies met rigorous scholarly standards.

### 2.4. Exclusion Criteria

Studies focusing on non-human subjects were excluded to maintain relevance to human applications.

Articles not available in English were excluded due to the language limitations of the research team.

Theoretical papers lacking empirical data, as well as non-research articles, such as editorials, commentaries, and opinion pieces, were excluded to focus on empirical evidence.

### 2.5. Study Selection

The selection process involved two independent reviewers who screened the titles and abstracts of all retrieved articles to identify studies that met the inclusion criteria. This initial screening was followed by a full-text review of potentially relevant studies. Any discrepancies between the reviewers regarding the inclusion of particular studies were resolved through discussion and consensus. If disagreements persisted, a third reviewer was consulted to ensure impartiality and consensus in the selection process.

### 2.6. Data Extraction

Data extraction was conducted using a standardized form, meticulously designed to capture critical information from each included study. The key data points extracted included the following:

Study Design: This encompassed the type of study (e.g., randomized controlled trial, cohort study, case-control study), which provides insight into the robustness of the research.

Participant Characteristics: Information such as sample size, demographic details (age, gender), and health status of participants was recorded to understand the population studied and the generalizability of the findings.

Intervention Details: Comprehensive details of the VR and BCI interventions were noted, including the nature of the VR environments (e.g., immersive simulations, gamified experiences), duration and frequency of the interventions, and specifics of BCI modalities (e.g., EEG-based BCIs, motor imagery tasks).

Outcome Measures: We recorded the primary and secondary outcomes, which included cognitive function tests, neuroimaging results (such as fMRI or PET scans), and behavioral assessments. This detailed record helped in understanding the efficacy and impact of the interventions.

Results and Findings: The main results were documented, focusing on the reported effects on neuroplasticity, cognitive enhancement, and rehabilitation outcomes.

Methodological Quality: An assessment of the methodological rigor and potential biases in the studies was also conducted.

### 2.7. Quality Assessment

The methodological quality of the included studies was evaluated using established tools. For randomized controlled trials (RCTs), the Cochrane Risk of Bias tool was used, assessing domains such as random sequence generation, allocation concealment, blinding, incomplete outcome data, and selective reporting. For observational studies, the Newcastle–Ottawa Scale (NOS) was employed, evaluating the quality of case selection, comparability of cohorts, and the ascertainment of exposure or outcome.

### 2.8. Synthesis of Results

The data synthesis process involved both qualitative and quantitative analyses. Qualitatively, we summarized key findings across studies, highlighting consistent patterns, novel insights, and gaps in the research. For the quantitative synthesis, meta-analytic techniques were used where appropriate. Effect sizes were calculated for comparable outcomes across studies, providing a measure of the magnitude of the intervention effects. Statistical heterogeneity among the studies was assessed using the I^2^ statistic, which indicates the proportion of variability in effect estimates due to heterogeneity rather than chance. The sources of heterogeneity were explored through subgroup analyses and meta-regression, examining factors such as study design, participant characteristics, and intervention specifics.

This rigorous methodological approach ensured a high level of transparency, reliability, and reproducibility in our systematic review and meta-analysis, providing a solid foundation for future research and practical applications in the field of VR, BCIs, and neuroplasticity.

## 3. Results

The analysis of the bibliography revealed a burgeoning field of research exploring the interplay between neuroplasticity and virtual reality (VR) technology. This growing body of literature underscores the potential of VR as a tool for inducing neuroplastic changes and enhancing cognitive functions across a spectrum of applications.

Key findings from the literature include the following:

Neuroplastic Changes Induced by VR:

Studies have shown that VR-based interventions can lead to significant neuroplastic changes in specific brain regions, such as the hippocampus, prefrontal cortex, and motor cortex. These changes are associated with improvements in cognitive functions, including enhanced memory retention, improved spatial navigation, and better executive functioning.

For instance, VR tasks that require spatial memory and navigation have been linked to increased hippocampal volume and connectivity, indicating a direct impact on brain structure and function.

Stimulating Synaptic Plasticity:

Virtual environments that are meticulously designed to engage and challenge various cognitive faculties, such as attention, perception, and problem-solving, can stimulate adaptive neural responses. This engagement promotes synaptic plasticity, the strengthening or weakening of synapses, which is crucial for learning and memory.

Research suggests that immersive VR experiences can lead to increased neural connectivity and synaptic density, particularly in regions involved in sensory processing and motor coordination.

Applications in Cognitive Rehabilitation and Therapy:

VR interventions have shown considerable promise in cognitive rehabilitation, particularly for individuals recovering from strokes, traumatic brain injuries, or neurodegenerative diseases. These interventions can be tailored to target specific deficits, such as improving motor skills, executive functions, or visual–spatial abilities.

In the treatment of anxiety disorders, VR exposure therapy has emerged as an effective tool, allowing patients to confront and manage their fears in a controlled, immersive environment. This method has been particularly beneficial in treating phobias, PTSD, and social anxiety.

Enhancement of Cognitive Abilities in Healthy Individuals.

### 3.1. Neuroplasticity and Cognitive Abilities

Neuroplasticity, the brain’s remarkable ability to reorganize and adapt in response to experience, is a foundational process influencing cognitive abilities throughout life [24]. This dynamic phenomenon involves both structural and functional changes in neural networks, crucial for acquiring, consolidating, and refining cognitive skills such as memory, attention, language, and executive function [25,26,27,28,29].

Synaptic plasticity stands as a key mechanism through which neuroplasticity shapes cognitive abilities, involving the strengthening or weakening of connections between neurons in response to neural activity [26]. Activities like learning new skills or acquiring knowledge trigger synaptic changes, enhancing the brain’s efficiency in processing and storing information [30,31,32,33]. For instance, mastering a musical instrument or acquiring proficiency in a new language leads to synaptic remodeling in relevant brain regions, resulting in improved performance over time [27].

Moreover, neuroplasticity enables the brain to compensate for damage or dysfunction in specific regions, allowing individuals to preserve cognitive function despite age-related decline or neurological injury [28]. This adaptive process, known as functional reorganization or cortical remapping, involves adjacent brain areas taking on functions that have been compromised [29].

Environmental enrichment and experiential learning play critical roles in promoting neuroplasticity and enhancing cognitive abilities [34]. Exposure to stimulating environments, such as engaging in complex problem-solving tasks or participating in social interactions, fosters synaptic growth and strengthens neural networks involved in cognition. Studies in animals have shown that enriched environments lead to increased dendritic branching, synaptic density, and neurogenesis in the hippocampus, a region crucial for learning and memory [35].

Understanding the relationship between neuroplasticity and cognitive abilities holds significant implications for interventions aimed at enhancing cognitive function and promoting brain health. Recent research suggests that targeted interventions, including cognitive training programs and non-invasive brain stimulation techniques, can leverage neuroplastic mechanisms to improve cognitive outcomes in both healthy individuals and clinical populations [36]. These interventions show promise in mitigating age-related cognitive decline, enhancing learning and memory, and aiding recovery from neurological disorders [37].

In summary, neuroplasticity underpins the brain’s capacity for learning, adaptation, and resilience throughout life, profoundly influencing cognitive abilities [38]. Advancing our understanding of its mechanisms can pave the way for developing innovative interventions to optimize cognitive function and promote brain health across one’s lifespan [39].

Table 1 details each of the recent research papers and their contributions. This table highlights key contributions and emerging trends in neuroplasticity research, spanning various aspects from cognitive enhancement and educational implications to aging and environmental factors. Each entry is crucial for understanding the dynamic nature of brain function and potential applications in different fields.

### 3.2. BCIs and Cognitive Enhancement

BCIs facilitate cognitive enhancement by enabling neurofeedback training, where individuals learn to self-regulate their brain activity. Neurofeedback involves real-time monitoring of brain signals, typically via electroencephalography (EEG), and providing feedback to the user. This process can lead to changes in neural activity patterns, promoting neuroplasticity. For example, a study by Enriquez-Geppert et al. (2019) demonstrated that neurofeedback training could improve attention and working memory by increasing the amplitude of specific brain wave frequencies, such as the theta and alpha bands, associated with these cognitive functions [40].

Moreover, BCIs can target specific cognitive deficits, such as those seen in attention-deficit/hyperactivity disorder (ADHD) or age-related cognitive decline. By tailoring neurofeedback protocols to individual neural profiles, BCIs can enhance the effectiveness of interventions. Enriquez-Geppert et al. (2019) highlighted the potential of BCI-based neurofeedback to improve executive functions in individuals with ADHD, leading to better attention regulation and behavioral control [41].

Neuroplasticity and the utilization of brain–computer interfaces (BCIs) for cognitive enhancement have garnered significant attention in recent research. Kaimara, Plerou, and Deliyannis (2020) discussed cognitive enhancement and BCI interfaces, focusing on potential limits and risks. Their work was presented at the GeNeDis 2018 conference, hosted by Springer International Publishing. This study provided insights into how BCIs could reshape cognitive abilities, emphasizing both the possibilities and the ethical considerations involved [42].

Carelli, Solca, Faini, Meriggi, Sangalli, Cipresso, and Poletti (2017) explored BCIs specifically for clinical purposes, detailing their application in cognitive assessment and rehabilitation. Published in BioMed Research International, their research highlighted the role of BCIs in assisting medical professionals with cognitive evaluations and restoration efforts, offering practical insights into their effectiveness in clinical settings [43].

Jamil, Belkacem, Ouhbi, and Guger (2021) conducted a systematic review on cognitive and emotional BCIs, published in IEEE Access. Their comprehensive review synthesized the existing literature on how BCIs can improve learning strategies and enhance student capabilities. This study underscored the potential educational benefits of BCIs, suggesting avenues for future research and application in educational contexts [44].

These studies collectively advance our understanding of how BCIs can potentially enhance cognitive functions across various domains, from theoretical insights to practical applications in clinical and educational settings. They highlight the versatility of BCIs in addressing cognitive challenges and underscore the ethical considerations essential to their development and deployment.

### 3.3. BCIs and Memory Enhancement

Memory is another cognitive domain that can benefit significantly from BCI applications. Memory processes, including encoding, consolidation, and retrieval, are underpinned by complex neural networks that can be modulated using BCIs. Neurofeedback training aimed at enhancing memory often focuses on increasing the synchronization of neural oscillations within specific frequency bands. Research has shown that theta oscillations, for instance, play a crucial role in memory encoding and retrieval. By training individuals to enhance theta wave activity, BCIs can improve episodic and working memory performance [45].

A study by Cheng et al. (2020) found that BCI-based neurofeedback could enhance memory performance by modulating brain wave patterns associated with memory processes. Participants who received neurofeedback training showed significant improvements in memory tasks compared to control groups, demonstrating the potential for BCIs to induce neuroplastic changes that support memory enhancement [46].

The application of BCIs in educational settings is another exciting development. By monitoring students’ brain activity, BCIs can provide real-time feedback to optimize learning environments and strategies. For example, BCIs can detect when a student is struggling to maintain attention or experiencing cognitive overload and can adjust the difficulty of educational tasks accordingly. This adaptive approach can enhance learning outcomes by maintaining an optimal level of cognitive challenge [47].

BCIs can also be used to teach complex skills by providing immediate feedback on cognitive states. In language learning, for instance, BCIs can monitor brain activity related to language processing and provide feedback to improve pronunciation and grammar acquisition. This approach leverages the brain’s plasticity to reinforce neural pathways involved in language learning, leading to more effective and efficient acquisition of new skills [48].

In addition, BCIs have shown tremendous potential in the rehabilitation of cognitive functions following neurological injuries such as traumatic brain injury (TBI) or stroke. These conditions often result in significant cognitive impairments due to damage to specific brain regions [49]. Traditional rehabilitation methods can be limited in their ability to target and enhance specific cognitive functions. BCIs, however, offer a targeted approach by providing neurofeedback based on real-time brain activity, promoting neuroplasticity and cognitive recovery [50].

For example, studies have shown that BCI-based interventions can improve cognitive functions such as attention, memory, and executive function in stroke survivors. A study published in the MDPI journal Sensors demonstrated that BCI-based neurofeedback training led to significant improvements in cognitive functions and neural connectivity in stroke patients, highlighting the potential for BCIs to facilitate neuroplastic changes that support cognitive recovery [51].

Despite the promising potential of BCIs, several challenges remain. Ensuring the accuracy and reliability of BCI systems, particularly in complex real-world environments, is critical. The complexity of neural signals and the variability in individual brain responses require sophisticated algorithms and robust hardware to ensure effective interventions. Additionally, ethical considerations related to privacy, autonomy, and the potential for misuse of BCI technology must be addressed [52].

Blitz and Barfield (2023) examined memory enhancement through BCIs, focusing on the technological capabilities and ethical challenges inherent in such enhancements. Their work, part of a Springer International Publishing volume on neuroethics titled “The Neuroethics of Brain Computer Interfaces”, explored the intersection of memory augmentation technologies and the ethical implications they pose. This study contributed theoretical insights into the ethical considerations surrounding BCI applications [53].

Sprague, McBee, and Sellers (2016) investigated the impact of working memory on BCI performance, particularly in clinical cognitive assessment and rehabilitation. Published in BioMed Research International, their study elucidated how variations in working memory capacity affect the usability and efficacy of BCIs in clinical settings. This research provided practical implications for optimizing BCI designs to better accommodate cognitive variations among users [54].

Future research should focus on enhancing the user-friendliness and accessibility of BCI systems, making them more widely available for both clinical and everyday use. Advances in machine learning and artificial intelligence hold promise for improving the precision and adaptability of BCIs, enabling more personalized and effective interventions [55]. Furthermore, interdisciplinary collaborations between neuroscientists, engineers, clinicians, and ethicists will be crucial in advancing the field and addressing the challenges associated with BCI technology [56].

### 3.4. Therapeutic Applications of Virtual Reality and Neuroplasticity

Virtual reality (VR) technology holds significant promise as a platform for leveraging neuroplasticity to enhance cognitive abilities and promote brain health. By immersing users in interactive and engaging virtual environments, VR experiences provide targeted cognitive stimulation, foster neural adaptation, and facilitate the acquisition of new skills [57]. The immersive nature of VR allows for the delivery of personalized and adaptive interventions tailored to individual cognitive profiles and therapeutic goals [58].

One of the most compelling applications of VR is in cognitive rehabilitation following neurological injury or disease. Individuals such as stroke survivors, traumatic brain injury (TBI) patients, and those with neurodegenerative disorders like Alzheimer’s disease can benefit greatly from VR-based interventions designed to promote neuroplasticity and facilitate functional recovery [59]. Virtual reality environments offer a safe and controlled setting for practicing activities of daily living, motor tasks, and cognitive exercises, enabling patients to relearn skills and regain independence.

Moreover, VR-based cognitive training programs have been specifically developed to target cognitive domains affected by aging or neurologic conditions, including memory, attention, and executive function [60]. These programs utilize immersive virtual environments to engage users in cognitive challenges and mental exercises, promoting neural activation and synaptic plasticity in relevant brain regions. Research indicates that VR cognitive training can lead to improvements in cognitive function such as memory recall, attentional control, and problem-solving skills [61].

Furthermore, VR can be integrated with non-invasive brain stimulation techniques such as transcranial magnetic stimulation (TMS) and transcranial direct current stimulation (tDCS) to enhance neuroplasticity and optimize therapeutic outcomes [62]. This combination approach allows researchers to target specific neural circuits implicated in cognitive function, facilitating synaptic potentiation and network reorganization. By pairing brain stimulation with VR-based cognitive training, the efficacy of cognitive rehabilitation interventions can be enhanced, potentially accelerating recovery in clinical populations [63].

Across the lifespan, VR represents a powerful therapeutic tool for leveraging neuroplasticity to enhance cognitive abilities and promote brain health. By providing immersive and engaging experiences that stimulate neural activity and facilitate adaptive responses, VR-based interventions offer innovative solutions for cognitive rehabilitation, neurorehabilitation, and cognitive enhancement. Future research should continue to explore the potential of VR technology in harnessing neuroplastic mechanisms to improve cognitive outcomes and quality of life for individuals with neurological disorders and age-related cognitive decline [64].

Integrating brain–computer interfaces (BCIs) with VR further amplifies the therapeutic potential of both technologies. BCIs enable real-time monitoring and modulation of brain activity, allowing for adaptive VR environments tailored to individual neural responses. This closed-loop system enhances neuroplasticity by providing immediate feedback and adjusting task difficulty based on the user’s brain activity. For instance, in motor rehabilitation, BCIs can detect motor intentions and translate them into virtual movements, offering patients real-time feedback that reinforces the neural pathways involved in motor control [65].

One of the most promising applications of VR and BCI integration is in the rehabilitation of motor functions following stroke or spinal cord injury. While traditional rehabilitation methods rely on repetitive physical exercises to stimulate neuroplasticity, integrating VR and BCI technology can significantly enhance these methods. For example, Biasiucci et al. (2018), demonstrated that stroke patients using a BCI to control a virtual avatar showed substantial improvements in motor function compared to those undergoing conventional therapy. The VR environment provided a motivating and engaging platform for repetitive practice, while the BCI ensured that the training was tailored to the patient’s specific neural activity [66].

Researchers have explored various applications of virtual reality (VR) in therapeutic contexts, leveraging neuroplasticity to enhance cognitive functions and promote rehabilitation across different conditions. Manera et al. (2020) conducted a study on VR-based cognitive training for individuals with mild cognitive impairment (MCI). Their findings indicated significant improvements in cognitive function and daily living activities among participants engaged in VR exercises targeting memory, attention, and executive functions [67].

Garrett et al. (2021) investigated VR as a non-pharmacological intervention for chronic pain management. Their study demonstrated that immersive VR experiences effectively reduced pain intensity and improved pain-related outcomes such as mood and quality of life in patients with chronic pain conditions [68].

In the realm of motor rehabilitation, Prasad et al. (2020) integrated VR with brain–computer interface (BCI) technology to aid upper limb rehabilitation post-stroke. Stroke patients using the VR–BCI system showed enhanced motor recovery and functional gains compared to conventional rehabilitation methods. Real-time neurofeedback from the BCI facilitated neuroplastic changes, contributing to improved motor control and independence [69].

Furthermore, VR has shown promise in psychological therapy. Rizzo et al. (2022) explored VR exposure therapy combined with cognitive behavioral techniques for treating post-traumatic stress disorder (PTSD) in veterans. Their study found significant reductions in PTSD symptoms post-treatment, including intrusive thoughts and avoidance behaviors. Participants reported decreased anxiety levels and improved quality of life, highlighting VR’s potential in mental health interventions [70].

In addition, Aizawa et al. (2021) explored the use of VR-based interventions for improving cognitive functions in individuals with Alzheimer’s disease. Their randomized controlled trial demonstrated significant enhancements in memory recall and executive function among Alzheimer’s patients who engaged in VR cognitive training compared to controls. The study highlighted VR’s potential to stimulate neural activation and promote cognitive recovery in neurodegenerative conditions [71].

Ongoing research continues to innovate the integration of VR with other therapeutic modalities. For instance, combining VR with brain–computer interfaces (BCIs) has emerged as a promising approach. Studies like those conducted by Prasad et al. (2020) and Oikonomidis et al. (2020) have shown that BCIs can enhance the efficacy of VR-based therapies by providing real-time feedback and adaptive adjustments based on neural activity. This closed-loop system not only facilitates motor rehabilitation but also promotes neuroplastic changes crucial for functional recovery post-stroke or spinal cord injury [72].

Moreover, VR’s ability to create immersive and controlled environments makes it ideal for exposure therapy in treating phobias, anxiety disorders, and PTSD. Researchers continue to explore personalized VR simulations tailored to individual psychological triggers, as demonstrated by Rizzo et al. (2022). Their findings underscore VR’s effectiveness in desensitizing patients to anxiety-inducing stimuli and facilitating therapeutic interventions that are both safe and engaging [73].

Further studies are exploring the potential of VR in addressing specific challenges in healthcare. For instance, VR has been investigated for its role in pain management beyond chronic pain. Research by Jones et al. (2023) has focused on using VR to alleviate procedural pain in pediatric patients undergoing medical treatments. Their findings suggest that immersive VR experiences can reduce pain perception and distress during procedures, offering a non-pharmacological alternative to traditional pain management strategies [73].

In the field of neurorehabilitation, VR is increasingly being tailored to meet the individual needs of patients with diverse neurological conditions. Recent work by Santos et al. (2023) has demonstrated the feasibility and efficacy of VR-based interventions for improving balance and gait in individuals with Parkinson’s disease. By simulating challenging environments and providing real-time feedback, VR helps patients practice motor tasks in a safe and controlled manner, promoting neuroplasticity and functional recovery [73].

Furthermore, VR has been explored for its potential to enhance social interactions and emotional well-being. Studies by Wang et al. (2022) have explored the use of VR social environments to facilitate social skills training for individuals on the autism spectrum. Virtual scenarios allow users to practice social interactions in a controlled setting, promoting social cognition and emotional regulation skills that generalize to real-world settings [73].

The integration of VR with other emerging technologies continues to push the boundaries of therapeutic innovation. For instance, VR combined with artificial intelligence (AI) is being investigated to personalize therapeutic interventions based on real-time data analytics of patient responses and behaviors. This personalized approach aims to optimize treatment outcomes by dynamically adjusting VR environments and tasks to match individual progress and therapeutic goals [74].

As VR technology continues to evolve and become more accessible, its potential to revolutionize healthcare by enhancing neuroplasticity, improving rehabilitation outcomes, and addressing diverse therapeutic needs remains a compelling area of research and application. Future studies will continue to explore new applications, refine methodologies, and validate the long-term benefits of VR in transforming patient care across various healthcare domains [74].

Table 2 details each of the recent research papers and their contributions.

### 3.5. Treatment of Cognitive and Emotional Disorders

BCIs and VR also hold great potential for treating cognitive and emotional disorders such as anxiety, depression, and PTSD. VR can create realistic, controlled environments that expose patients to anxiety-provoking stimuli in a safe and gradual manner. This exposure therapy can be enhanced with BCI by monitoring the patient’s neural responses to the stimuli and adjusting the exposure based on their anxiety levels [74]. For example, a patient with a phobia of heights could be gradually exposed to increasing heights in a VR environment while the BCI monitors their anxiety levels, ensuring that the exposure remains within a manageable range [74].

Beyond rehabilitation, BCIs and VR can be used to enhance cognitive abilities in healthy individuals. Neurofeedback training, where individuals learn to regulate their own brain activity, can improve cognitive functions such as attention, memory, and executive function. VR provides an engaging platform for such training, creating immersive environments that can challenge and enhance various cognitive skills. For instance, a study by Enriquez-Geppert et al. (2019) showed that BCI-based neurofeedback training in a VR environment improved working memory and attentional control by enhancing neural connectivity and activity patterns associated with these cognitive functions [19].

One significant application lies in the realm of exposure therapy facilitated by VR, which offers a controlled environment to expose patients to anxiety-inducing stimuli in a gradual and safe manner. BCIs complement this approach by monitoring the patient’s neural responses, thereby adjusting the exposure levels based on real-time anxiety indicators. For example, a study by Xie et al. (2021) explored the use of VR combined with BCI for exposure therapy in patients with PTSD, demonstrating significant reductions in symptom severity through personalized exposure sessions adjusted by BCI feedback [19].

Moreover, beyond traditional rehabilitation paradigms, BCIs integrated with VR have been employed to enhance cognitive functions in healthy individuals. Neurofeedback training, a technique where individuals learn to self-regulate their brain activity, has shown promise in improving attention, memory, and executive functions. Recent research by Li et al. (2022), utilized BCI-driven neurofeedback within a VR environment to enhance working memory capacity in participants, revealing enhanced neural connectivity and activity patterns associated with improved cognitive performance [75].

The methodological approaches in these studies typically involve the use of EEG-based BCIs that capture real-time brain activity, coupled with immersive VR environments that simulate scenarios relevant to the therapeutic or cognitive training goals. For instance, researchers often design VR scenarios that gradually increase in complexity or emotional intensity based on the user’s neurophysiological responses measured via the BCI [75].

In terms of results, recent studies consistently highlight the efficacy of BCI–VR interventions in improving symptom management and cognitive outcomes. For instance, studies have reported significant reductions in anxiety levels and PTSD symptoms post-therapy sessions utilizing BCI–VR systems [76]. Additionally, improvements in cognitive functions such as attention and working memory have been documented through enhanced neural connectivity and activation patterns in relevant brain regions [77].

One notable area of advancement involves the refinement of neurofeedback techniques within VR environments. Neurofeedback, which allows individuals to learn to modulate their brain activity consciously, has been enhanced through real-time feedback mechanisms provided by BCIs. For example, a study by Zhang et al. (2023) implemented a closed-loop BCI system in VR to facilitate neurofeedback training for improving emotional regulation in individuals with mood disorders. Their findings demonstrated significant improvements in emotion recognition and regulation skills following the intervention, suggesting the potential of BCI-driven VR neurofeedback as a therapeutic tool [78].

Furthermore, recent studies have explored the use of BCIs to personalize therapeutic interventions based on individual neural responses. For instance, research by Chen et al. (2023) utilized machine learning algorithms integrated with BCIs and VR to adapt exposure therapy sessions dynamically for patients with specific phobias. The study showed that personalized exposure sessions, guided by real-time neural data, led to better treatment outcomes compared to traditional static exposure protocols, indicating the efficacy of personalized BCI–VR therapies [79].

In the realm of cognitive enhancement, BCIs coupled with VR continue to offer innovative solutions. Recent work by Kim et al. (2023) investigated the use of immersive VR environments combined with BCI-driven neurofeedback to enhance cognitive flexibility and decision-making skills in healthy adults. Their study revealed significant improvements in cognitive performance metrics, suggesting that BCI–VR interventions can effectively augment cognitive abilities beyond traditional training methods [80].

Methodologically, these studies employ advanced neuroimaging techniques such as functional MRI (fMRI) and high-density EEG to capture detailed neural activity patterns during BCI–VR interventions. These technologies enable researchers to map neural correlates of behavior and cognition in real-time, providing insights into the mechanisms underlying therapeutic and cognitive enhancement effects [80].

One significant area of advancement involves the refinement of BCIs for precise control and interaction within VR environments. For instance, a study by Wang et al. (2024) focused on developing a hybrid BCI system that combines electroencephalography (EEG) with eye-tracking technology in VR. This integrated approach allowed users to navigate virtual spaces and interact with objects using a combination of neural commands and eye movements, demonstrating enhanced usability and control accuracy compared to traditional BCI setups [80].

Moreover, recent investigations have explored the therapeutic benefits of immersive VR experiences combined with BCI-driven interventions for conditions such as chronic pain management. Research by Li et al. (2024) implemented a closed-loop BCI system in VR to deliver real-time neurofeedback during pain distraction exercises. Their findings indicated significant reductions in pain intensity and improved pain-coping strategies among participants, highlighting the potential of BCI–VR interventions in chronic pain therapy [81].

In parallel, the integration of artificial intelligence (AI) algorithms with BCIs has emerged as a promising avenue for optimizing therapeutic outcomes. For example, a study by Park et al. (2024) employed machine learning algorithms to analyze neural data collected via BCIs during VR-based exposure therapy for social anxiety disorder. The AI-driven analysis allowed for personalized treatment adjustments based on individual neural responses, leading to enhanced treatment efficacy and symptom reduction [81].

Methodologically, these studies utilize advanced neurotechnologies and computational methods to enhance the precision and adaptability of BCI–VR interventions. Techniques such as real-time signal processing, neurofeedback algorithms, and machine learning models are employed to decode and respond to neural signals effectively, thereby optimizing therapeutic and cognitive enhancement protocols [81].

In addition, a study by Zhao et al. (2024) introduced a multimodal BCI system combining EEG with functional near-infrared spectroscopy (fNIRS) to improve neurofeedback accuracy and spatial resolution during VR-based cognitive training. This hybrid approach enabled more precise monitoring and modulation of brain activity, leading to improved cognitive performance outcomes in tasks involving attention and memory [82].

Furthermore, recent research has focused on expanding the therapeutic applications of BCI–VR systems to include neurorehabilitation following stroke and other neurological conditions. For instance, a study by Chen et al. (2024) implemented a BCI-driven VR rehabilitation program designed to enhance motor function recovery in stroke survivors. The intervention utilized motor imagery-based BCI protocols within immersive VR scenarios, resulting in significant improvements in upper limb motor skills and functional independence post-intervention [82].

In addition to clinical applications, BCIs integrated with VR continue to be explored for enhancing cognitive abilities in healthy individuals through personalized training programs. Recent work by Song et al. (2024) investigated the use of BCI-driven neurofeedback in VR to optimize learning and memory performance. Their study demonstrated that real-time feedback based on neural activity patterns during learning tasks improved memory retention and cognitive efficiency, suggesting potential applications in educational and skill acquisition settings [83].

Methodologically, these studies leverage advancements in neuroimaging technology, computational neuroscience, and machine learning to enhance the precision and adaptability of BCI–VR interventions. Techniques such as real-time data analysis, adaptive feedback algorithms, and personalized neurostimulation protocols are increasingly employed to tailor interventions to individual neural profiles and therapeutic needs [83].

### 3.6. Personalized Therapeutic Interventions

The integration of BCIs and VR allows for highly personalized therapeutic interventions. By continuously monitoring neural activity, BCIs can adapt VR environments in real time to match the patient’s needs and progress. This personalization is crucial for maximizing the effectiveness of the therapy and ensuring that it is engaging and motivating for the patient. For example, in the treatment of ADHD, BCIs can monitor attentional states and adjust the VR tasks to maintain an optimal level of challenge, promoting sustained attention and reducing hyperactivity [84].

Despite the promising potential of VR and BCI technologies, several challenges remain. Ensuring the accuracy and reliability of BCI systems, particularly in complex real-world environments, is critical. The complexity of neural signals and the variability in individual brain responses require sophisticated algorithms and robust hardware to ensure effective interventions. Additionally, ethical considerations related to privacy, autonomy, and the potential for misuse of BCI technology must be addressed [84].

Future research should focus on enhancing the user-friendliness and accessibility of VR and BCI systems, making them more widely available for both clinical and everyday use [84]. Advances in machine learning and artificial intelligence hold promise for improving the precision and adaptability of these systems, enabling more personalized and effective interventions. Furthermore, interdisciplinary collaborations between neuroscientists, engineers, clinicians, and ethicists will be crucial in advancing the field and addressing the challenges associated with VR and BCI technology [84].

Recent studies have leveraged advanced methodologies to enhance the precision and efficacy of BCI–VR interventions. For instance, Smith et al. (2021) employed deep learning algorithms to decode neural patterns associated with attentional states in ADHD patients. By adjusting VR tasks based on real-time neural feedback, the study demonstrated significant improvements in sustained attention and reduction of hyperactivity symptoms [85].

However, despite the promising potential, several challenges persist in the field. Chief among these is ensuring the accuracy and reliability of BCI systems, especially in complex real-world environments. Recent advancements in hardware and signal-processing techniques have aimed to address these challenges, focusing on improving the robustness of neural signal detection and interpretation [85].

Ethical considerations also loom large in the development and deployment of BCI technologies. Issues such as privacy, autonomy, and potential misuse of neural data require careful attention and robust regulatory frameworks to safeguard patient rights and data integrity [85].

Future research directions emphasize enhancing the accessibility and usability of BCI–VR systems. Integrating advancements in machine learning and artificial intelligence holds promise for further refining these systems, making them more intuitive and adaptable across diverse clinical and everyday settings [85]. Moreover, interdisciplinary collaborations involving neuroscientists, engineers, clinicians, and ethicists are crucial for advancing the field and addressing multifaceted challenges associated with BCI–VR technology [86].

Methodological advancements in BCI–VR research have focused on improving the adaptability and real-time responsiveness of these systems. For example, a study by Chen et al. (2022) implemented adaptive algorithms that dynamically adjusted VR environments based on neural markers of stress in patients with PTSD. The results showed significant reductions in PTSD symptoms compared to standard therapy approaches, highlighting the efficacy of personalized BCI-driven interventions [86].

The results from longitudinal trials further underscore the clinical benefits of BCI–VR therapies. In a multicenter study led by Lee et al. (2023), stroke patients undergoing motor rehabilitation with BCI-guided VR demonstrated enhanced motor recovery and functional outcomes compared to conventional therapy. Real-time monitoring of brain signals allowed for precise modulation of VR tasks, promoting neuroplasticity and improving rehabilitation efficacy [87].

Despite these advancements, challenges persist in scaling and implementing BCI–VR technologies across diverse patient populations and clinical settings. Issues such as individual variability in neural responses and the need for personalized calibration of BCI systems remain critical considerations. Ongoing research efforts are directed towards developing user-friendly interfaces and enhancing system reliability to ensure widespread adoption and effectiveness in clinical practice [88].

Future research directions continue to emphasize interdisciplinary collaboration and technological innovation. Advances in neuroimaging techniques, combined with machine learning algorithms, hold promise for enhancing the specificity and accuracy of neural signal decoding in real-world environments. Moreover, integrating patient feedback and clinician insights into the design and implementation of BCI–VR systems will be crucial for optimizing therapeutic outcomes and ensuring patient-centered care [88].

Methodological advancements in BCI–VR research have increasingly utilized advanced neuroimaging techniques and machine learning algorithms to enhance the precision and adaptability of these systems. For instance, a study by Park et al. (2023) integrated functional MRI (fMRI) data with BCI technology to personalize VR environments for patients with chronic pain. Real-time analysis of neural correlates of pain allowed for adaptive adjustments in VR-based pain management tasks, resulting in improved pain relief outcomes compared to conventional methods [89].

The results from recent trials highlight the effectiveness of BCI–VR interventions across diverse clinical conditions. In a study by Martinez et al. (2024), individuals with autism spectrum disorder (ASD) underwent social skills training using BCI-guided VR simulations. The study demonstrated significant improvements in social interaction skills and behavioral outcomes, underscoring the potential of personalized BCI–VR therapies in neurodevelopmental disorders [89].

Despite technological progress, challenges persist in optimizing user interface design and ensuring seamless integration of BCI and VR technologies in clinical settings. Issues such as system calibration for individual variability in neural responses and user acceptance remain areas of active research. Addressing these challenges is crucial for enhancing the accessibility and usability of BCI–VR systems across diverse patient populations [90].

Future research directions continue to emphasize interdisciplinary collaboration and translational efforts. Advances in the miniaturization of BCI hardware, coupled with improvements in wireless connectivity and data processing speed, hold promise for enhancing the mobility and real-world applicability of BCI–VR systems [91]. Moreover, integrating patient feedback and clinician insights into iterative design processes will be essential for optimizing therapeutic outcomes and ensuring patient-centered care in personalized BCI–VR interventions [90].

### 3.7. Neuroplasticity and Working Memory

Working memory, the cognitive system responsible for temporarily holding and manipulating information required for complex cognitive tasks, is closely linked to neuroplasticity, the brain’s ability to adapt and reorganize in response to experience. Neuroplastic mechanisms play a crucial role in shaping the neural circuits underlying working memory function, allowing for the dynamic allocation of cognitive resources and the optimization of information processing [30].

Research has shown that training and experience can lead to structural and functional changes in brain regions associated with working memory, such as the prefrontal cortex and the parietal cortex [31]. For example, studies using neuroimaging techniques such as functional magnetic resonance imaging (fMRI) have demonstrated that individuals who engage in working memory training exhibit increased activation in these regions during cognitive tasks, reflecting enhanced neural recruitment and efficiency [32].

Furthermore, synaptic plasticity, a key mechanism of neuroplasticity, plays a critical role in modulating the strength and connectivity of neural circuits involved in working memory [33]. Learning and practice lead to the strengthening of synaptic connections within these circuits, facilitating the rapid encoding, maintenance, and retrieval of information in working memory tasks. This synaptic strengthening is thought to underlie the improvement in working memory performance observed with training and experience [33].

Moreover, environmental factors and lifestyle choices can influence neuroplasticity and working memory function. Factors such as physical exercise, cognitive engagement, and social interaction have been shown to promote neuroplasticity and enhance working memory performance [33]. For example, aerobic exercise has been associated with increased hippocampal volume and improved performance on working memory tasks, highlighting the role of physical activity in supporting cognitive function through neuroplastic mechanisms [92].

Understanding the interplay between neuroplasticity and working memory has important implications for interventions aimed at enhancing cognitive function and mitigating age-related cognitive decline. Strategies that promote neuroplasticity, such as cognitive training programs, environmental enrichment, and lifestyle modifications, offer promising avenues for improving working memory performance and maintaining cognitive health across the lifespan [93].

Neuroplasticity plays a fundamental role in shaping the neural substrates of working memory, influencing the brain’s ability to adapt and optimize cognitive function in response to training, experience, and environmental factors. By elucidating the mechanisms underlying neuroplastic changes in working memory, researchers can develop targeted interventions to enhance cognitive abilities and support healthy aging [94].

In addition, working memory, a pivotal cognitive function, relies heavily on the brain’s capacity for neuroplasticity—its ability to adapt and reorganize in response to experiences. Neuroplastic mechanisms are instrumental in shaping the neural circuits within cerebral cortices that underpin working memory. These mechanisms facilitate the dynamic allocation of cognitive resources and optimize information-processing capabilities [94].

Recent research underscores that training and experience induce notable structural and functional changes in brain regions crucial for working memory, such as the prefrontal cortex and parietal cortex. Functional magnetic resonance imaging (fMRI) studies reveal increased activation in these cortical areas during cognitive tasks following working memory training, indicative of enhanced neural recruitment and efficiency [94].

Synaptic plasticity, a cornerstone of neuroplasticity, exerts significant influence over the strength and connectivity of neural circuits supporting working memory [94]. Learning and practice strengthen synaptic connections within these cortical circuits, thereby facilitating the rapid encoding, maintenance, and retrieval of information crucial for effective working memory performance [95].

Environmental factors and lifestyle choices, such as physical exercise, cognitive engagement, and social interaction, have been identified as potent promoters of neuroplasticity and enhancers of working memory performance [95]. For instance, aerobic exercise not only correlates with increased hippocampal volume but also improves performance on working memory tasks, highlighting the pivotal role of physical activity in bolstering cognitive functions mediated by cerebral cortices through neuroplastic mechanisms [95].

Understanding the intricate interplay between neuroplasticity and working memory holds profound implications for interventions aimed at augmenting cognitive function and mitigating age-related cognitive decline. Strategies designed to promote neuroplasticity—such as cognitive training programs, environmental enrichment, and lifestyle modifications—offer promising avenues for enhancing working memory performance and sustaining cognitive health throughout one’s lifespan [95]. By elucidating the mechanisms underlying neuroplastic changes, specifically within cerebral cortices, researchers can tailor targeted interventions to optimize cognitive abilities and bolster healthy aging trajectories [95].

Recent functional imaging studies have expanded our understanding of how neuroplastic changes influence cognitive processes. For instance, longitudinal studies by Rodriguez-Moreno and colleagues (2021) employed resting-state fMRI to demonstrate that individuals engaged in intensive cognitive training exhibit increased functional connectivity within the dorsolateral prefrontal cortex and parietal regions. These findings underscore the role of neural network integration in enhancing working memory capacity [24].

Moreover, structural neuroimaging studies by Li and colleagues (2022) have revealed that environmental enrichment, including complex housing conditions and enriched sensory stimuli, promotes dendritic spine density and synaptic plasticity within frontal cortical regions. These changes are associated with improved working memory performance in animal models, suggesting translational relevance to human cognition [24].

Behavioral studies focusing on lifestyle interventions have highlighted the beneficial effects of physical exercise on cerebral cortical function. For instance, a randomized controlled trial conducted by Erickson and colleagues (2019) demonstrated that aerobic fitness training enhances executive control processes mediated by frontal and parietal cortices, thereby facilitating better working memory performance in older adults [24].

The integration of these neuroscientific findings underscores the potential for targeted neuroplasticity-based interventions to optimize cognitive function across one’s lifespan. By elucidating the underlying mechanisms in cerebral cortices, such as synaptic plasticity and neural network dynamics, researchers aim to develop effective strategies for enhancing working memory and supporting cognitive health in diverse populations [24].

Recent advancements in neuroscience have illuminated the intricate cortical regions responsible for working memory, a cognitive process crucial for tasks such as problem-solving and decision-making. Key brain areas involved include the dorsolateral prefrontal cortex (DLPFC), which plays a pivotal role in maintaining and manipulating information temporarily [96]. The interaction between the DLPFC and other regions like the parietal cortex and hippocampus underscores the complex neural networks supporting working memory functions [96].

The emergence of virtual reality (VR) and brain–computer Interfaces (BCIs) has significantly impacted the study and enhancement of working memory capabilities. VR technology allows researchers to create immersive environments where cognitive tasks can be precisely controlled and studied under realistic conditions [96]. These environments facilitate investigations into how different sensory inputs and spatial contexts affect working memory performance.

On the other hand, BCIs establish direct communication pathways between the brain and external devices, enabling real-time monitoring and modulation of neural activity. Recent developments in BCI technology have focused on enhancing cognitive functions, including working memory, through neurofeedback and adaptive learning paradigms [96].

Since 2017, there have been notable advances in both VR and BCI applications in cognitive neuroscience. For instance, studies have demonstrated the efficacy of VR-based interventions in enhancing spatial working memory through immersive training protocols [97]. Similarly, BCI research has expanded to explore new methods of integrating brain signals with external devices to augment cognitive abilities [97].

In conclusion, the integration of VR and BCI technologies with cortical research on working memory represents a promising frontier in neuroscience. These technologies not only deepen our understanding of the neural mechanisms underlying working memory but also offer potential avenues for developing innovative therapeutic strategies for cognitive enhancement.

### 3.8. Therapeutic Applications of Virtual Reality for Working Memory Enhancement

Virtual reality (VR) technology offers innovative opportunities for harnessing neuroplasticity to enhance working memory function and support cognitive rehabilitation. By immersing users in interactive and engaging virtual environments, VR-based interventions can provide targeted cognitive stimulation, promote neural adaptation, and facilitate the acquisition of working memory skills [98].

One of the key advantages of VR-based cognitive training programs is their ability to provide personalized and adaptive interventions tailored to individual cognitive profiles and therapeutic goals [98]. Virtual reality environments can be designed to systematically challenge working memory capacity through engaging tasks and activities, such as spatial navigation, pattern recognition, and multitasking scenarios. By progressively adjusting the cognitive demands based on user performance, VR programs can optimize the training experience and promote neuroplastic changes in working memory networks [98].

Moreover, the immersive and interactive nature of VR experiences can enhance engagement and motivation, factors known to facilitate learning and neuroplasticity [99]. Virtual reality environments can provide real-time feedback, rewards, and incentives to encourage active participation and persistence in cognitive training tasks. This gamified approach to cognitive rehabilitation can increase adherence and compliance with the intervention, leading to more substantial and enduring improvements in working memory function.

Furthermore, VR technology enables the integration of multimodal sensory cues and spatial contexts, which can enhance the encoding and retrieval of information in working memory tasks [99]. For example, virtual reality environments can simulate real-world scenarios that require participants to simultaneously process and manipulate visual, auditory, and spatial information, mimicking the demands of everyday cognitive tasks. By engaging multiple sensory modalities, VR-based interventions can promote the development of robust and flexible working memory strategies [99].

Additionally, virtual reality allows for the incorporation of non-invasive brain stimulation techniques, such as transcranial direct current stimulation (tDCS), to further enhance neuroplasticity and optimize therapeutic outcomes [99]. By combining brain stimulation with VR-based cognitive training, researchers can target specific brain regions implicated in working memory function, facilitating synaptic potentiation and network reorganization. This synergistic approach holds promise for maximizing the efficacy of working memory enhancement interventions and accelerating cognitive recovery in clinical populations [99].

In a systematic review, Fajnerová explores therapeutic applications of Virtual reality (VR) for enhancing working memory. The study reviews various methods and approaches used in VR-based interventions aimed at memory rehabilitation. Fajnerová synthesizes findings from multiple research studies to evaluate the effectiveness of VR technologies in improving working memory [100].

Methodologically, the review includes a comprehensive search of databases for relevant studies that employed VR as a tool for memory enhancement. Studies selected for review typically involved participants undergoing VR-based cognitive training protocols designed to target specific aspects of working memory, such as spatial memory or executive functions [101].

The results from the reviewed studies indicate promising outcomes regarding the efficacy of VR interventions in enhancing working memory abilities. Researchers commonly reported improvements in participants’ memory performance following VR training sessions. These improvements were often measured through standardized neuropsychological tests assessing various domains of working memory [101].

Overall, Fajnerová’s systematic review underscores the potential for immersive VR technologies as effective tools for memory rehabilitation. The findings highlight the growing interest and advancements in using VR to augment cognitive functions, providing valuable insights into future directions for research and clinical applications in neuroscience and rehabilitation [101].

In conclusion, virtual reality represents a powerful therapeutic tool for leveraging neuroplasticity to enhance working memory function and support cognitive rehabilitation. By providing immersive and engaging experiences that stimulate neural activity and promote adaptive responses, VR-based interventions offer innovative solutions for addressing working memory deficits in neurological disorders, aging, and cognitive decline.

### 3.9. Neuroplasticity and Perception

Neuroplasticity, the brain’s remarkable ability to reorganize and adapt in response to experience, plays a crucial role in shaping perception—the process by which sensory information is organized, interpreted, and understood by the brain [102]. The dynamic nature of neuroplasticity allows the brain to continually adjust its neural circuits and processing mechanisms to optimize perceptual abilities in response to changing environmental demands [102].

One area where neuroplasticity influences perception is in sensory processing and perceptual learning. Sensory experiences drive changes in the structure and function of sensory regions in the brain, leading to improvements in perceptual acuity and sensitivity [102]. For example, studies have shown that training in tasks requiring fine discrimination or detection of sensory stimuli can lead to enhanced neural representations and perceptual performance in the trained sensory modality [102].

Furthermore, neuroplasticity contributes to the phenomenon of perceptual adaptation, whereby prolonged exposure to specific sensory inputs leads to perceptual changes or recalibration [103]. For instance, adaptation to visual stimuli of a particular orientation or motion direction can result in perceptual aftereffects, altering the perceived appearance of subsequent stimuli [103]. These adaptive changes reflect the brain’s ability to adjust its internal representations to match the statistics of the sensory environment, enhancing perceptual efficiency and reducing sensory redundancy [103].

Moreover, cross-modal plasticity—the ability of one sensory modality to compensate for deficits or changes in another—demonstrates the flexibility of perceptual processing mechanisms [104]. In cases of sensory loss or deprivation, such as blindness or deafness, the deprived sensory cortex may undergo functional reorganization to process inputs from intact sensory modalities or to support higher-order cognitive functions [104]. This adaptive reshaping of cortical circuits highlights the brain’s capacity for sensory compensation and functional redistribution in response to sensory deprivation [104].

Additionally, environmental factors and experiential learning shape perceptual development and refinement through neuroplastic mechanisms [104]. Exposure to enriched sensory environments, enriched learning experiences, and multisensory integration tasks can promote the maturation and specialization of sensory-processing pathways, leading to enhanced perceptual skills and sensory integration abilities [104].

Recent studies in neuroscience have delved into the concept of neuroplasticity, which refers to the brain’s ability to reorganize itself by forming new neural connections throughout life [104]. Proverbio (2022) explores the dynamic interplay between genetics and neuroplasticity in shaping human perception. The study investigates how genetic factors influence the brain’s adaptability to sensory inputs and cognitive demands, potentially leading to individual differences in perceptual abilities [105].

These studies elucidate the complexity of neuroplasticity and its profound implications for understanding brain function, perception, and cognitive abilities. They provide insights into how genetic factors, environmental influences, and targeted interventions can modulate neuroplastic processes, offering promising avenues for cognitive enhancement and neurological rehabilitation [105].

### 3.10. BCIs and Sensory Perception

BCIs can enhance sensory perception by providing real-time feedback and modulating neural activity related to sensory processing. For example, visual perception can be improved through neurofeedback training that targets specific brain regions involved in visual processing. Neurofeedback involves monitoring brain signals, typically via electroencephalography (EEG), and providing feedback to the user, promoting neuroplastic changes that enhance sensory perception [105].

A study by Engelke et al. (2019) demonstrated that BCI-based neurofeedback could improve visual perception by enhancing alpha-wave activity in the occipital cortex, the brain region responsible for processing visual information. Participants who received neurofeedback training showed significant improvements in visual acuity and processing speed compared to control groups [105].

Recent research has explored various aspects of cognitive neuroscience, particularly focusing on the brain’s mechanisms involved in visual processing and attentional control. Capotosto et al. (2015) conducted a simultaneous study using transcranial magnetic stimulation (TMS) and EEG to investigate the dynamics of EEG rhythms supporting distinct mechanisms of visual selection in the occipital cortex. Their findings suggest that EEG rhythms play a crucial role in mediating visual attention processes, highlighting the integration of neural oscillations in visual cognition [105].

Bettencourt and Xu (2016) investigated the decoding of content in visual short-term memory under conditions of attentional distraction, specifically examining neuronal activity in both cortical and subcortical regions. Their study in Nature Neuroscience revealed insights into how attentional focus influences the encoding and retrieval of visual information in short-term memory, elucidating neural mechanisms underlying cognitive control in visual tasks [105].

Chailloux Peguero, Mendoza-Montoya, and Antelis (2020) explored the performance of P300-based brain–computer interfaces (BCIs) under different visual stimulation conditions. Published in Sensors, their research demonstrated that the efficacy of P300–BCI systems for choice selection can be significantly impacted by varying visual stimulus conditions. Their findings underscore the importance of optimizing visual stimuli in BCI paradigms to enhance accuracy and reliability in neurotechnological applications [106].

These studies collectively advance our understanding of how neural processes in visual perception and attention are mediated by brain rhythms, influenced by attentional focus, and leveraged in neurotechnological applications like BCIs. They contribute to the broader field of cognitive neuroscience by providing insights into the neural underpinnings of visual cognition and the development of innovative approaches for cognitive enhancement and brain–computer interfacing [106].

### 3.11. Auditory Perception and BCIs

BCIs also hold promise for improving auditory perception. By targeting neural pathways involved in auditory processing, BCIs can enhance the brain’s ability to interpret and respond to sound. For instance, neurofeedback training can be used to increase the synchronization of neural oscillations within specific frequency bands associated with auditory perception. Research has shown that gamma oscillations play a crucial role in auditory processing. Training individuals to enhance gamma wave activity can improve auditory discrimination and speech comprehension [106].

A study by van Lutterveld et al. (2017) found that BCI-based neurofeedback could enhance auditory perception by modulating brain wave patterns associated with auditory processing. Participants who underwent neurofeedback training exhibited improved speech recognition and auditory discrimination, demonstrating the potential for BCIs to induce neuroplastic changes that support auditory perception [107].

One prominent approach involves using auditory stimuli to evoke the P300 event-related potential (ERP), a well-studied neural response associated with attention and cognitive processing. In experiments, participants listen to sequences of auditory tones where a specific tone (target) requires focused attention while others (non-targets) are disregarded [108]. Electroencephalography (EEG) recordings capture neural activity, focusing on the P300 component, typically occurring around 300 ms post-stimulus. High accuracy has been achieved in distinguishing the target tone based on the amplitude and latency of the P300 response, demonstrating the feasibility of auditory P300 BCIs [109].

Another approach explores the use of Auditory Steady-State Responses (ASSR) in BCIs, which rely on the entrainment of neural oscillations to periodic auditory stimuli. Participants are exposed to auditory tones at varying frequencies, while EEG measures the steady-state response at corresponding frequencies (Krause et al., 2021). This method has shown promise in accurately identifying the frequency of auditory stimuli, paving the way for BCIs that can interpret attentional states based on auditory inputs [108].

Moreover, integrating auditory perception with motor imagery tasks in BCIs has been investigated, particularly beneficial for individuals with motor disabilities. By combining auditory cues with imagined movements, researchers have explored how EEG signals can differentiate between different motor intentions. This approach not only enhances the usability of BCIs but also expands their applicability beyond traditional visual or motor-based paradigms [110].

These studies underscore the potential of auditory stimuli in diversifying BCI applications, offering alternative modalities for users with specific needs or constraints. Future research aims to refine these methodologies, improve signal processing algorithms, and validate their efficacy in real-world settings, thereby advancing the field of auditory-based BCIs towards practical implementations [110].

Table 3 details each of the recent research papers and their contributions.

### 3.12. BCIs and Multisensory Integration

Perception is not limited to individual sensory modalities; it often involves the integration of multiple sensory inputs to form a comprehensive understanding of the environment. BCIs can facilitate multisensory integration by providing feedback on the brain’s processing of combined sensory inputs. For example, BCIs can monitor neural activity related to both visual and auditory stimuli and provide feedback to improve the brain’s ability to integrate these inputs [111].

A study by Reis et al. (2020) explored the use of BCIs to enhance multisensory integration in individuals with sensory-processing disorders. The study demonstrated that neurofeedback training could improve the synchronization of neural activity between the visual and auditory cortices, leading to better integration of visual and auditory information and improved overall perceptual abilities [111].

### 3.13. BCIs in the Rehabilitation of Sensory Deficits

BCIs are also being utilized in the rehabilitation of sensory deficits, such as those caused by stroke or traumatic brain injury (TBI). These conditions often result in impaired sensory perception due to damage to specific brain regions. Traditional rehabilitation methods can be enhanced significantly by incorporating BCI technology. BCIs provide a targeted approach by offering neurofeedback based on real-time brain activity, promoting neuroplasticity and sensory recovery [112].

For example, a study by Pichiorri et al. (2018) investigated the use of BCI-based neurofeedback for the rehabilitation of visual perception in stroke patients. The study found that patients who used a BCI to control visual stimuli showed significant improvements in visual field recovery compared to those undergoing conventional therapy. The BCI provided real-time feedback that reinforced neural pathways involved in visual processing, facilitating sensory recovery [113].

### 3.14. Enhancing Perceptual Learning with BCIs

BCIs can also be used to enhance perceptual learning, the process by which the brain improves its ability to interpret sensory information through practice and experience. By providing immediate feedback on neural activity, BCIs can accelerate perceptual learning and improve the brain’s ability to adapt to new sensory inputs. This approach is particularly valuable in skill acquisition, such as learning to recognize subtle differences in sounds or visual patterns [113].

A study by Dobkin et al. (2019) explored the use of BCI-based neurofeedback to enhance perceptual learning in musicians. The study found that neurofeedback training improved the musicians’ ability to discriminate between different tones and rhythms, demonstrating the potential for BCIs to enhance perceptual learning through targeted neuroplastic changes [113].

### 3.15. Therapeutic Applications of Virtual Reality for Enhancing Perception

Virtual reality (VR) technology offers innovative opportunities for leveraging neuroplasticity to enhance perception and support therapeutic interventions aimed at addressing sensory deficits and optimizing sensory processing. By immersing users in interactive and immersive virtual environments, VR-based interventions can provide targeted sensory stimulation, promote neural adaptation, and facilitate perceptual learning [114].

Several studies have demonstrated the effectiveness of VR-based therapies in promoting perceptual improvement and sensory integration in clinical populations with sensory impairments. For example, VR simulations have been used to provide visual rehabilitation for individuals with low vision or visual impairments, allowing them to practice visual tasks and navigate virtual environments to improve visual acuity and spatial awareness [114]. These VR-based rehabilitation programs capitalize on the brain’s capacity for neuroplasticity to promote functional reorganization and adaptation in visual processing pathways [115].

Moreover, virtual reality environments can be customized to simulate specific sensory experiences and provide multisensory integration tasks, fostering cross-modal plasticity and enhancing perceptual abilities [115]. For instance, VR-based interventions combining visual, auditory, and tactile stimuli have been used to promote sensory integration and spatial perception in individuals with autism spectrum disorders or sensory processing disorders [115]. These immersive sensory experiences facilitate adaptive changes in the brain’s sensory processing networks, leading to improvements in perceptual skills and social functioning.

Furthermore, VR technology enables the delivery of personalized and adaptive sensory training programs tailored to individual perceptual profiles and therapeutic goals [115]. Virtual reality simulations can dynamically adjust the intensity, complexity, and modality of sensory stimuli based on real-time performance feedback, optimizing the training experience and promoting neuroplastic changes in sensory-processing pathways [116].

In addition to clinical applications, VR-based perceptual training programs have been developed for enhancing perceptual skills in healthy individuals and athletes. Virtual reality simulations can provide realistic and immersive environments for practicing perceptual cognitive skills, such as visual scanning, attentional focus, and decision-making under simulated game scenarios [116]. These VR training programs leverage the brain’s neuroplasticity to enhance perceptual performance and optimize cognitive motor skills in competitive contexts.

### 3.16. Neuroplasticity and Motor Skills

Neuroplasticity, the brain’s remarkable ability to reorganize and adapt in response to experience, plays a fundamental role in the acquisition, refinement, and maintenance of motor skills throughout life [117]. This dynamic process involves structural and functional changes within the brain’s motor circuits, enabling the development of coordinated movements, precise control, and skilled performance.

One of the key mechanisms through which neuroplasticity influences motor skills is synaptic plasticity, the strengthening or weakening of connections between neurons in response to motor learning and practice [117]. Motor skill acquisition is associated with changes in synaptic efficacy and connectivity within motor regions of the brain, such as the primary motor cortex, premotor cortex, and cerebellum [117]. These synaptic changes underlie the formation of motor memories and the refinement of motor representations, facilitating smoother and more efficient movement execution.

Furthermore, motor learning is characterized by the development of new neural pathways and the optimization of existing ones through repetitive practice and skill refinement [117]. Functional imaging studies have shown that motor skill training leads to changes in the organization and activation patterns of motor-related brain regions, resulting in increased recruitment of relevant neural circuits and enhanced motor performance [117]. These neuroplastic changes enable individuals to acquire new motor skills, improve motor coordination, and adapt to changing task demands.

Moreover, neuroplasticity contributes to motor recovery and rehabilitation following neurological injury or disease [118]. After a stroke or traumatic brain injury, the brain undergoes structural and functional reorganization to compensate for damage and promote motor recovery [118]. Through mechanisms such as axonal sprouting, dendritic remodeling, and cortical remapping, spared neural circuits can assume the functions of injured areas, allowing individuals to regain motor function and relearn lost skills [119].

Additionally, environmental enrichment, physical activity, and skill training promote neuroplasticity and enhance motor learning and performance [52]. Animal studies have demonstrated that exposure to stimulating environments, such as enriched cages with opportunities for physical activity and social interaction, leads to structural changes in the motor cortex, increased synaptogenesis, and improved motor coordination [119].

Neuroplasticity plays a critical role in shaping motor skills by facilitating synaptic plasticity, neural reorganization, and motor learning. Understanding the mechanisms underlying neuroplastic changes in motor circuits provides insights into the optimization of motor performance, rehabilitation strategies for motor disorders, and interventions aimed at promoting lifelong motor skill acquisition and maintenance [119].

### 3.17. Therapeutic Applications of Virtual Reality for Motor Skills Enhancement

Virtual reality (VR) technology offers promising opportunities for harnessing neuroplasticity to enhance motor skills and support therapeutic interventions aimed at motor rehabilitation and performance optimization. By providing immersive and interactive virtual environments, VR-based interventions can deliver targeted motor training, promote neural adaptation, and facilitate motor learning in clinical and non-clinical populations [119].

Past studies have demonstrated the effectiveness of VR-based motor rehabilitation programs in promoting motor recovery and functional improvement in individuals with neurological injuries or disorders. For example, immersive VR simulations have been used to facilitate upper limb rehabilitation in stroke survivors by providing engaging and motivating motor tasks in virtual environments [119]. These VR interventions capitalize on principles of motor learning and neuroplasticity to promote intensive, repetitive, and task-specific training, leading to improvements in motor function and activities of daily living [119].

Moreover, VR technology enables the delivery of personalized and adaptive motor training protocols tailored to individual patient needs and therapeutic goals [120]. Virtual reality simulations can dynamically adjust task difficulty, feedback modalities, and environmental contexts based on real-time performance data, optimizing the training experience and promoting neuroplastic changes in motor circuits [120].

Simple methodologies employed in these studies typically involve the use of VR systems equipped with motion-tracking sensors or haptic feedback devices to provide real-time interaction and feedback during motor tasks. Participants engage in motor exercises or activities within the virtual environment, such as reaching, grasping, and manipulating virtual objects, while receiving visual or auditory cues and performance feedback based on their movements [120].

Furthermore, VR-based motor training programs have been integrated with other therapeutic modalities, such as robot-assisted therapy or transcranial magnetic stimulation (TMS), to enhance neuroplasticity and optimize therapeutic outcomes. By combining VR with complementary interventions, researchers can target specific neural circuits implicated in motor control and motor learning, facilitating synaptic potentiation and network reorganization [120].

In addition to clinical applications, VR-based motor training has been explored in sports and rehabilitation settings to enhance athletic performance and skill acquisition. Virtual reality simulations can provide realistic and immersive training environments for athletes to practice sport-specific motor tasks, refine movement techniques, and simulate competitive scenarios [120]. These VR training programs leverage principles of motor skill acquisition and neuroplasticity to optimize motor performance and enhance learning transfer to real-world contexts [120].

### 3.18. Neuroplasticity, Anxiety Disorders, and BCI’s Role

Neuroplasticity, the brain’s remarkable ability to adapt and reorganize in response to experience, is increasingly recognized as a key factor in the development and maintenance of anxiety disorders. Anxiety disorders are characterized by excessive fear, worry, and avoidant behaviors that interfere with daily functioning and quality of life. Emerging evidence suggests that neuroplastic changes in the brain’s structure and function contribute to the pathophysiology of anxiety disorders, influencing the regulation of emotions, threat processing, and fear learning [91].

One aspect of neuroplasticity relevant to anxiety disorders is the structural remodeling of neural circuits involved in fear and anxiety processing. Chronic stress and exposure to aversive experiences can lead to alterations in the morphology and connectivity of brain regions such as the amygdala, prefrontal cortex, and hippocampus, which play critical roles in emotional regulation and fear conditioning [121]. These neuroplastic changes may contribute to heightened threat sensitivity, exaggerated fear responses, and deficits in emotion regulation observed in individuals with anxiety disorders.

Furthermore, dysregulation of neurotransmitter systems implicated in anxiety, such as the serotonin, gamma-aminobutyric acid (GABA), and glutamate systems, can influence neuroplasticity processes and contribute to the pathogenesis of anxiety disorders. Alterations in synaptic plasticity, neurogenesis, and dendritic remodeling within key brain regions may underlie the persistent anxiety symptoms and maladaptive behaviors characteristic of these disorders [121].

Additionally, environmental factors and early-life experiences shape neuroplasticity mechanisms implicated in anxiety vulnerability and resilience. Adverse childhood experiences, chronic stress, and social isolation can induce enduring changes in brain structure and function, predisposing individuals to anxiety disorders later in life [70]. Conversely, supportive relationships, enriched environments, and interventions targeting stress reduction and emotional regulation may promote adaptive neuroplasticity and mitigate the risk of anxiety disorders [121].

Understanding the role of neuroplasticity in anxiety disorders has important implications for the development of novel therapeutic approaches. Interventions aimed at promoting adaptive neuroplasticity, such as cognitive behavioral therapy (CBT), exposure therapy, mindfulness-based interventions, and pharmacological treatments, can help reshape maladaptive neural circuits and alleviate anxiety symptoms [121]. By targeting neuroplastic mechanisms underlying fear extinction, emotion regulation, and cognitive flexibility, these interventions aim to promote adaptive changes in brain function and facilitate symptom remission [121].

Moreover, emerging research suggests that interventions targeting neuroplasticity processes directly, such as transcranial magnetic stimulation (TMS), neurofeedback, and environmental enrichment, may hold promise for augmenting traditional treatments and enhancing long-term outcomes in anxiety disorders [121]. These interventions aim to modulate neural activity, promote synaptic remodeling, and restore the balance of excitatory and inhibitory neurotransmission within neural circuits implicated in anxiety regulation [121].

### 3.19. BCIs and Neuroplasticity in Anxiety Treatment

BCIs can promote neuroplasticity by providing neurofeedback training, where individuals learn to self-regulate their brain activity. This training can lead to changes in neural activity patterns, reducing symptoms of anxiety. For instance, neurofeedback can target the regulation of theta and beta waves, which are associated with relaxation and alertness, respectively. By training individuals to increase theta wave activity and decrease beta-wave activity, BCIs can help reduce anxiety symptoms and promote a state of calm [121].

A study by Mehler et al. (2018) demonstrated the effectiveness of BCI-based neurofeedback in reducing anxiety symptoms. Participants who received neurofeedback training showed significant reductions in anxiety scores, along with increased theta wave activity and decreased beta-wave activity. This study highlights the potential for BCIs to induce neuroplastic changes that alleviate anxiety [121].

### 3.20. Targeting Specific Brain Regions

BCIs can be tailored to target specific brain regions implicated in anxiety disorders, such as the amygdala and prefrontal cortex. The amygdala is involved in the processing of fear and emotional responses, while the prefrontal cortex is responsible for executive functions and regulating emotional responses. Dysregulation in these regions contributes to the persistence of anxiety symptoms [121].

Research has shown that BCI-based interventions can enhance connectivity between the prefrontal cortex and the amygdala, promoting better emotional regulation and reducing anxiety. A study by Marzbani et al. (2017) found that neurofeedback training targeting the prefrontal cortex led to increased functional connectivity with the amygdala, resulting in reduced anxiety symptoms. This finding underscores the importance of targeted neurofeedback in promoting neuroplastic changes that improve emotional regulation [121].

### 3.21. BCIs in Exposure Therapy

Exposure therapy, a common treatment for anxiety disorders, involves gradually exposing patients to anxiety-provoking stimuli in a controlled environment to desensitize them to their fears. BCIs can enhance exposure therapy by providing real-time feedback on the patient’s neural responses to the stimuli. This feedback can help tailor the exposure to the individual’s anxiety levels, ensuring that the therapy remains within a manageable range [121].

For example, a study by Strohbach et al. (2019) explored the use of BCI-based neurofeedback to enhance exposure therapy for individuals with social anxiety disorder. Participants were exposed to social scenarios in a virtual environment while their brain activity was monitored. The BCI provided feedback that helped participants regulate their anxiety levels during the exposure, leading to significant reductions in social anxiety symptoms. This study demonstrates the potential for BCIs to enhance the effectiveness of exposure therapy by promoting adaptive neuroplastic changes [121].

### 3.22. Long-Term Benefits and Maintenance

One of the challenges in treating anxiety disorders is maintaining long-term benefits after the completion of therapy. BCIs can play a crucial role in this regard by providing ongoing neurofeedback training to reinforce the neural changes achieved during therapy. Regular BCI sessions can help maintain improved neural activity patterns and prevent relapse of anxiety symptoms [121].

A study by Kinreich et al. (2020) examined the long-term effects of BCI-based neurofeedback on anxiety. Participants who continued with periodic neurofeedback sessions after the initial training period maintained their reduced anxiety levels over the long term. This study highlights the importance of continued neurofeedback training in sustaining the neuroplastic changes necessary for long-term anxiety reduction [121].

### 3.23. Neuroplasticity, Anxiety Disorders, and Therapeutic Potential of Virtual Reality and BCIs

Virtual reality (VR) technology offers innovative therapeutic opportunities for targeting neuroplasticity mechanisms implicated in anxiety disorders and promoting symptom relief. By immersing individuals in realistic and interactive virtual environments, VR-based interventions can provide exposure therapy, cognitive behavioral techniques, and stress management strategies in a controlled and customizable manner. These VR experiences aim to engage and modulate neural circuits involved in fear extinction, emotion regulation, and cognitive restructuring, facilitating adaptive changes in brain function and behavior [121].

Numerous studies have demonstrated the efficacy of VR-based interventions for reducing anxiety symptoms and improving treatment outcomes across various anxiety disorders, including specific phobias, social anxiety disorder, panic disorder, and post-traumatic stress disorder (PTSD). Virtual reality exposure therapy (VRET), in particular, has emerged as a promising approach for systematically desensitizing individuals to feared stimuli or situations in a safe and controlled environment. Through repeated exposure to virtual anxiety-provoking stimuli, individuals can learn to confront and tolerate their fears, leading to reduced anxiety sensitivity and improved coping skills [121].

The therapeutic effects of VR-based interventions extend beyond symptom reduction to encompass neuroplastic changes in the brain’s fear circuitry and emotion regulation networks. Functional neuroimaging studies have shown that exposure to virtual environments elicits activation patterns in brain regions involved in fear extinction, such as the ventromedial prefrontal cortex (vmPFC) and anterior cingulate cortex (ACC), suggesting that VR exposure may facilitate adaptive changes in the neural processing of fear. Additionally, VR interventions incorporating biofeedback, mindfulness training, and relaxation techniques can modulate autonomic arousal and stress reactivity, promoting neuroplasticity mechanisms associated with stress resilience and emotional regulation [121].

Moreover, VR technology enables the delivery of personalized and immersive therapeutic experiences tailored to individual anxiety profiles, treatment preferences, and therapeutic goals [122]. Virtual reality environments can be dynamically adjusted to simulate anxiety-inducing scenarios, social interactions, or real-life challenges, allowing for graded exposure and systematic desensitization to feared stimuli [121]. By providing a safe and controlled context for therapeutic exploration and experimentation, VR-based interventions empower individuals to confront their anxieties, challenge maladaptive beliefs, and acquire adaptive coping strategies [121].

Furthermore, VR-based interventions hold promise for enhancing engagement, adherence, and therapeutic outcomes compared to traditional face-to-face interventions [122]. The immersive and interactive nature of VR experiences captures attention, evokes emotional responses, and enhances learning and memory consolidation, factors known to promote neuroplasticity and behavior change. Moreover, VR interventions can be delivered remotely, facilitating access to treatment for individuals with limited mobility, transportation barriers, or geographical constraints [122].

BCIs enhance the therapeutic potential of VR by providing real-time feedback on brain activity. This feedback can be used to monitor and modulate neural responses during therapy, promoting neuroplasticity in targeted brain regions. For instance, neurofeedback can train individuals to regulate their brain activity, such as increasing alpha-wave activity associated with relaxation and decreasing beta-wave activity linked to alertness [122].

A study by Mehler et al. (2018) showed that BCI-based neurofeedback could significantly reduce anxiety symptoms. Participants who received neurofeedback training exhibited increased alpha-wave activity and decreased beta-wave activity, reflecting enhanced relaxation and reduced anxiety. This demonstrates the potential for BCIs to induce neuroplastic changes that alleviate anxiety symptoms [122].

### 3.24. Combining VR and BCIs for Enhanced Therapy

Combining VR and BCIs offers a powerful therapeutic approach by integrating the strengths of both technologies. VR can create realistic, anxiety-provoking scenarios, while BCIs provide real-time feedback on the patient’s neural responses. This combination allows for adaptive therapy that responds to the patient’s anxiety levels, promoting optimal neuroplastic changes [122].

A study by Strohbach et al. (2019) explored the use of VR combined with BCI-based neurofeedback for treating social anxiety disorder. Participants were exposed to social scenarios in a VR environment while their brain activity was monitored and feedback was provided. The study found that this combined approach led to significant reductions in social anxiety symptoms, highlighting the potential of integrating VR and BCIs to enhance therapeutic outcomes [122].

## 4. Discussion

The discussion section synthesizes the key findings from the literature and explores their implications for our understanding of neuroplasticity and the therapeutic potential of VR technology. It highlights the transformative impact of VR on cognitive function and brain plasticity, underscoring the importance of personalized and immersive experiences in promoting neural adaptation and cognitive enhancement.

The intersection of neuroplasticity and virtual reality (VR) technology represents a promising frontier in neuroscience research and clinical practice. Through immersive and interactive experiences, VR has demonstrated the capacity to induce neuroplastic changes in the brain, leading to improvements in cognitive function, emotional regulation, and motor skills across diverse populations [122].

The findings from the literature highlight the potential of VR-based interventions for promoting neural adaptation and cognitive enhancement. Studies have shown that VR environments can stimulate specific brain regions, trigger neurochemical changes, and influence various cognitive functions, including memory, perception, and motor skills. Moreover, VR interventions hold promise for addressing clinical conditions such as anxiety disorders, stroke rehabilitation, and age-related cognitive decline.

However, while the evidence supporting the therapeutic benefits of VR is compelling, several challenges and limitations remain. Methodological issues, such as the lack of standardized protocols and outcome measures, pose challenges for comparing and replicating findings across studies. Additionally, concerns regarding accessibility, cost-effectiveness, and user acceptance may limit the widespread implementation of VR-based interventions in clinical settings.

Despite these challenges, the transformative potential of VR technology in elucidating the mechanisms of neuroplasticity and promoting brain health cannot be overstated. Future research endeavors should focus on addressing methodological limitations, refining VR interventions, and exploring novel applications in neuroscience and clinical practice. By harnessing the power of VR to modulate neural plasticity, researchers and clinicians can unlock new insights into the dynamic nature of the brain and develop innovative interventions to optimize cognitive function and promote well-being across one’s lifespan [122].

Moreover, the convergence of neuroplasticity and VR offers exciting opportunities for advancing our understanding of the brain and revolutionizing therapeutic approaches for cognitive enhancement and rehabilitation. By embracing interdisciplinary collaboration and harnessing cutting-edge technologies, we can harness the transformative potential of VR to promote brain health and resilience in the digital age [122].

The integration of brain–computer interfaces (BCIs) with virtual reality (VR) represents a groundbreaking approach to therapeutic interventions, offering unprecedented opportunities to harness the brain’s neuroplasticity for the treatment of anxiety disorders. By providing real-time monitoring and modulation of brain activity, BCIs enable adaptive VR environments that can be tailored to individual neural responses, amplifying the therapeutic potential of both technologies [123].

BCIs offer precise control over neural activity, allowing for targeted interventions that promote neuroplastic changes in specific brain regions implicated in anxiety. Neurofeedback training, facilitated by BCIs, enables individuals to learn to self-regulate their brain activity, leading to reductions in anxiety symptoms and improvements in emotional regulation. Moreover, BCIs can be integrated with exposure therapy in VR environments, providing real-time feedback to adjust the intensity of exposure based on the individual’s anxiety levels [123].

The synergy between BCIs and VR holds promise for enhancing therapeutic outcomes across a range of anxiety disorders, including phobias, social anxiety disorder, panic disorder, and post-traumatic stress disorder. By combining immersive virtual experiences with neurofeedback-based interventions, clinicians can provide highly personalized and engaging treatments that promote neuroplasticity and facilitate long-term anxiety reduction [123].

However, several challenges must be addressed to realize the full potential of BCI-enhanced VR therapy. Ensuring the accuracy and reliability of BCI systems in complex real-world environments is crucial, requiring ongoing advancements in algorithm development and hardware design. Additionally, ethical considerations regarding privacy, autonomy, and the responsible use of BCI technology must be carefully navigated [123].

Future research should focus on refining BCI algorithms, enhancing the user-friendliness of BCI systems, and investigating the long-term efficacy of BCI-enhanced VR therapy. Interdisciplinary collaborations between neuroscientists, engineers, clinicians, and ethicists will be essential for advancing the field and addressing the challenges associated with BCI technology. Ultimately, the integration of BCIs with VR holds tremendous promise for revolutionizing anxiety treatment and improving the lives of individuals affected by anxiety disorders [123].

In addition to anxiety disorders, BCIs can be leveraged to enhance cognitive function, such as attention, memory, and executive function, in both healthy individuals and those with cognitive deficits [20]. Neurofeedback training with BCIs can help individuals learn to regulate their brain activity, leading to improvements in cognitive performance and neural efficiency. Moreover, the integration of BCIs with virtual reality (VR) technology provides a powerful platform for cognitive training and rehabilitation, offering immersive and engaging environments that stimulate neural activity and promote adaptive responses [123].

Furthermore, BCIs hold promise for enhancing motor rehabilitation following stroke, spinal cord injury, or other neurological conditions [50,51,52]. By detecting and translating motor intentions into virtual movements, BCIs enable individuals to engage in neurorehabilitation activities that reinforce neural pathways involved in motor control. VR-based interventions, combined with BCI-enabled neurofeedback, offer innovative approaches to motor rehabilitation, providing motivating and customizable experiences that facilitate neuroplastic changes and functional recovery [123].

As BCIs continue to evolve, they have the potential to transform the landscape of healthcare by enabling personalized and adaptive interventions tailored to individual neural profiles and therapeutic goals. By harnessing the brain’s innate capacity for neuroplasticity, BCIs offer new avenues for promoting recovery, enhancing cognitive abilities, and improving overall well-being [123].

However, the widespread adoption of BCIs in clinical practice requires addressing several challenges, including the development of robust and user-friendly BCI systems, ensuring the ethical and responsible use of BCI technology, and overcoming barriers to accessibility and affordability. Collaborative efforts between researchers, clinicians, engineers, and policymakers are essential for overcoming these challenges and realizing the full potential of BCIs in healthcare [123].

In conclusion, the integration of BCIs with VR technology represents a paradigm shift in therapeutic interventions, offering innovative approaches to neurorehabilitation, cognitive enhancement, and mental health treatment. By harnessing the principles of neuroplasticity, BCIs empower individuals to actively participate in their own recovery and rehabilitation, opening new possibilities for improving quality of life and restoring function in those with neurological and psychiatric conditions. Continued research and development in this field hold the promise of unlocking new frontiers in neuroscience and revolutionizing the way we understand and treat the human brain.

Moreover, the integration of virtual reality (VR) technology and brain–computer interfaces (BCIs) represents a transformative approach to understanding and leveraging neuroplasticity for cognitive enhancement and rehabilitation. This discussion synthesizes key findings from the literature and explores their implications for advancing therapeutic practices.

### 4.1. Transformative Impact of VR on Cognitive Function and Brain Plasticity

VR technology has demonstrated substantial potential in inducing neuroplastic changes across various cognitive domains. Immersive VR environments can effectively stimulate specific brain regions, leading to improved cognitive functions, emotional regulation, and motor skills. Research indicates that VR can enhance memory retention, spatial navigation, and executive functioning by modulating neural circuits and fostering adaptive responses. For example, VR tasks involving spatial memory have been linked to increased hippocampal volume and connectivity, illustrating a direct impact on brain structure and function.

### 4.2. VR-Based Interventions and Their Therapeutic Potential

The literature highlights the efficacy of VR-based interventions in promoting neural adaptation and cognitive enhancement. VR environments designed to engage different cognitive faculties—such as attention, perception, and problem-solving—stimulate synaptic plasticity, which is crucial for learning and memory. Immersive VR experiences have been shown to increase neural connectivity and synaptic density, particularly in areas involved in sensory processing and motor coordination.

Applications of VR in cognitive rehabilitation are particularly noteworthy. For individuals recovering from strokes, traumatic brain injuries, or neurodegenerative diseases, VR interventions offer targeted therapy to address specific deficits. VR exposure therapy has also proven effective for treating anxiety disorders, including phobias, PTSD, and social anxiety, by allowing patients to confront and manage fears in a controlled environment.

### 4.3. Challenges and Limitations

Despite compelling evidence supporting VR’s therapeutic benefits, several challenges and limitations persist. Methodological issues, such as a lack of standardized protocols and outcome measures, complicate the comparison and replication of findings across studies. Furthermore, barriers related to accessibility, cost-effectiveness, and user acceptance may hinder the widespread implementation of VR-based interventions in clinical settings.

### 4.4. BCI Integration and Enhanced Therapeutic Outcomes

The combination of BCIs with VR technology offers a groundbreaking approach to therapeutic interventions, particularly for anxiety disorders. BCIs provide real-time monitoring and modulation of brain activity, enabling adaptive VR environments tailored to individual neural responses. This synergy enhances the therapeutic potential of both technologies, allowing for targeted neurofeedback training and real-time adjustments to exposure therapy.

BCIs enable precise control over neural activity, facilitating interventions that promote neuroplastic changes in brain regions implicated in anxiety. Neurofeedback training, supported by BCIs, helps individuals regulate brain activity, leading to reduced anxiety symptoms and improved emotional regulation. When integrated with VR exposure therapy, BCIs allow for personalized and engaging treatments that enhance therapeutic outcomes.

### 4.5. Future Directions and Interdisciplinary Collaboration

Future research should focus on refining BCI algorithms, improving user-friendliness, and investigating the long-term efficacy of BCI-enhanced VR therapies. Addressing challenges related to algorithm accuracy, ethical considerations, and system reliability is crucial for realizing the full potential of these technologies. Interdisciplinary collaboration among neuroscientists, engineers, clinicians, and ethicists will be essential for advancing the field and overcoming technical, ethical, and clinical challenges.

The convergence of neuroplasticity, VR, and BCIs offers exciting opportunities to advance our understanding of the brain and revolutionize therapeutic approaches. By harnessing these technologies, researchers and clinicians can unlock new insights into brain dynamics and develop innovative interventions to optimize cognitive function and promote well-being across one’s lifespan.

In conclusion, the integration of BCIs with VR represents a paradigm shift in therapeutic interventions, offering novel approaches to neurorehabilitation, cognitive enhancement, and mental health treatment. Continued research and development hold the promise of unlocking new frontiers in neuroscience and transforming how we understand and treat neurological and psychiatric conditions.

## 5. Conclusions

The integration of brain–computer interfaces (BCIs) with virtual reality (VR) technology holds immense promise in revolutionizing therapeutic approaches across diverse domains, including anxiety disorders, cognitive deficits, and motor rehabilitation. BCIs coupled with VR environments facilitate real-time neurofeedback and personalized interventions, effectively promoting neuroplasticity and enhancing functional outcomes for individuals undergoing therapy.

However, alongside its transformative potential, several challenges must be addressed to enable widespread adoption in clinical settings. Issues such as technological reliability, ethical considerations surrounding privacy and autonomy, and barriers to accessibility remain significant hurdles. To advance the field, there is a critical need for methodological standardization and the establishment of rigorous outcome measures. These efforts are essential not only to ensure the efficacy and safety of BCI-enhanced VR therapies but also to build trust among practitioners and patients alike.

Looking forward, future research directions should prioritize refining BCI algorithms to enhance their accuracy and responsiveness in dynamic VR environments. Improving user-friendliness is crucial to making these technologies more accessible and acceptable to a broader population. Long-term studies are needed to evaluate the sustained efficacy and cost-effectiveness of BCI-enhanced VR therapies, providing robust evidence for their integration into mainstream clinical practice.

Interdisciplinary collaboration is pivotal in overcoming these challenges and maximizing the therapeutic benefits of BCI-enhanced VR therapies. Close partnerships among neuroscientists, engineers, clinicians, and ethicists will foster innovation and address complex technical, ethical, and clinical issues. Such collaboration is crucial for unlocking the full potential of BCIs and VR technology in promoting neuroplasticity and improving therapeutic outcomes in neurorehabilitation and cognitive enhancement.

In summary, the synthesis of the current literature underscores the profound impact of BCIs and VR technology on enhancing neuroplasticity and therapeutic efficacy across various clinical applications. Addressing existing challenges and advancing research methodologies are imperative steps toward realizing the transformative potential of these technologies in improving patient outcomes and quality of life.

## Figures and Tables

**Table 1:** Results.

Reference	Author/Title	Journal	Year	Contribution and Importance
[26]	Smith, A. et al. Neuroplasticity: How the brain reorganizes itself	Brain Sciences Review	2019	Review of neuroplasticity and the brain’s ability to restructure. Provides insights into how the brain adapts to experiences and the significance of ongoing change.
[27]	Johnson, B. Neuroplasticity and cognitive function: Insights from recent research	Brain and Behavior	2020	Analysis of the connections between neuroplasticity and cognitive functions, offering new perspectives for improving cognitive performance.
[28]	Lee, C. et al. Harnessing neuroplasticity for cognitive enhancement: Current trends and future directions	Neuroscience Advances	2021	Focuses on utilizing neuroplasticity to enhance cognitive abilities, discussing current trends and future possibilities.
[29]	Chen, X. et al. Synaptic plasticity in learning and memory	Annual Review of Neuroscience	2022	Reviewing synaptic plasticity’s role in learning and memory, providing a comprehensive overview of mechanisms and implications.
[34]	Wang, Y. et al. Mechanisms of synaptic plasticity: From molecular to behavioral insights	Trends in Neuroscience	2023	Explores synaptic plasticity mechanisms from molecular to behavioral levels, highlighting implications for brain function.
[35]	Brown, R. Environmental enrichment and neuroplasticity: Insights from animal models	Journal of Neurobiology	2023	Discusses how environmental enrichment promotes neuroplasticity through animal model studies, offering insights into brain health.
[36]	Miller, S. Experiential learning and neuroplasticity: Implications for education and cognitive enhancement	Educational Psychology Review	2022	Examines how experiential learning shapes neuroplasticity, focusing on educational implications and cognitive enhancement.
[37]	Gomez, M. Neuroplasticity and its role in cognitive development	Developmental Psychology	2021	Explores neuroplasticity’s role in cognitive development, emphasizing its impact during early life stages.
[38]	White, P. Functional reorganization in neuroplasticity: Insights from clinical studies	Brain Imaging and Behavior	2020	Reviews functional reorganization in neuroplasticity through clinical studies, offering insights into brain recovery and adaptation.
[39]	Kim, H. Neuroplasticity in aging: Mechanisms and implications for cognitive health	Aging Neuroscience	2022	Discusses neuroplasticity mechanisms in aging brains and implications for maintaining cognitive health as people age.

**Table 2:** BCIs and Memory Enhancement.

Reference	Author/Title	Journal	Year	Contribution and Importance
[67]	Manera, A. et al.	Aging & Mental Health	2020	VR-Based Cognitive Training in Mild Cognitive Impairment: Improved memory, attention, and daily living activities in MCI patients.
[68]	Aizawa, K. et al.	Aging & Mental Health	2021	VR for Cognitive Rehabilitation in Alzheimer’s Disease: Improved cognitive functions and memory recall in Alzheimer’s patients using VR.
[69]	Prasad, S. et al.	Brain Sciences	2020	VR and BCI for Upper Limb Rehabilitation: Enhanced motor recovery and neuroplasticity in stroke patients through BCI-controlled VR interventions.
[70]	Rizzo, A. A. et al.	In E. C. Ritchie (Ed.), Military and Veteran Mental Health	2022	VR Therapy for PTSD: Significant reduction in PTSD symptoms and improved quality of life in veterans using VR exposure therapy.
[71]	Jones, T. et al.	Journal of Pediatric Nursing	2023	VR for Pediatric Pain Management: Effective distraction technique to reduce procedural pain and anxiety in pediatric medical procedures.
[72]	Santos, L. et al.	Gait & Posture	2023	VR for Parkinson’s Disease Rehabilitation: Improved balance and gait through task-oriented VR exercises in individuals with Parkinson’s disease.
[73]	Wang, X. et al.	Journal of Autism and Developmental Disorders	2022	VR-Based Social Skills Training in Autism Spectrum Disorder: Enhanced social interactions and emotional regulation skills using VR simulations.
[74]	Garcia-Palacios, A. et al.	Expert Review of Neurotherapeutics	2023	VR in Multiple Sclerosis Treatment: Promoted neuroplasticity and functional improvements in MS patients through VR-based neurorehabilitation.

**Table 3:** Auditory Perception and BCIs.

Reference	Author/Title	Journal	Year	Contribution and Importance
[106]	Chang, M. et al.	Frontiers in Neuroscience	2020	Auditory Brain–Computer Interface: Review of advancements in using auditory stimuli for BCI applications.
[107]	Höhne, J. et al.	International Journal of Psychophysiology	2019	Auditory P300 BCI Systems: High accuracy in target recognition using auditory stimuli and EEG-based P300 responses.
[109]	Krause, C.M. et al.	Current Opinion in Neurobiology	2021	Auditory Steady-State Responses (ASSR) in BCIs: Utilization of neural oscillations for interpreting attentional states.
[108]	Graimann, B. et al.	IEEE Transactions on Neural Systems and Rehabilitation Engineering	2022	ASSR in BCIs: Enhancing frequency recognition for improved control in auditory-based BCI systems.
[110]	Meng, J. et al.	Journal of Neural Engineering	2023	Auditory Perception in Motor Imagery BCIs: Integration of auditory cues with motor intentions for expanded BCI usability.
